# Factors influencing the performance of community health workers: A qualitative study of Anganwadi Workers from Bihar, India

**DOI:** 10.1371/journal.pone.0242460

**Published:** 2020-11-25

**Authors:** Aparna John, Nicholas Nisbett, Inka Barnett, Rasmi Avula, Purnima Menon

**Affiliations:** 1 Department of International Development, University of Oxford, Oxford, United Kingdom; 2 Health and Nutrition Cluster, Institute of Development Studies, University of Sussex, Brighton, United Kingdom; 3 Poverty, Health and Nutrition Division, International Food Policy Research Institute, New Delhi, India; The University of Sydney Faculty of Medicine and Health, AUSTRALIA

## Abstract

Globally, there remain significant knowledge and evidence gaps around how to support Community Health Worker (CHW) programmes to achieve high coverage and quality of interventions. India’s Integrated Child Development Services scheme employs the largest CHW cadre in the world—Anganwadi Workers (AWWs). However, factors influencing the performance of these workers remain under researched. Lessons from it have potential to impact on other large scale global CHW programmes. A qualitative study of AWWs in the Indian state of Bihar was conducted to identify key drivers of performance in 2015. In-depth interviews were conducted with 30 AWWs; data was analysed using both inductive and deductive thematic analysis. The study adapted and contextualised existing frameworks on CHW performance, finding that factors affecting performance occur at the individual, community, programme and organisational levels, including factors not previously identified in the literature. Individual factors include initial financial motives and family support; programme factors include beneficiaries’ and AWWs’ service preferences and work environment; community factors include caste dynamics and community and seasonal migration; and organisational factors include corruption. The initial motives of the worker (the need to retain a job for family financial needs) and community expectations (for product-oriented services) ensure continued efforts even when her motivation is low. The main constraints to performance remain factors outside of her control, including limited availability of programme resources and challenging relationships shaped by caste dynamics, seasonal migration, and corruption. Programme efforts to improve performance (such as incentives, working conditions and supportive management) need to consider these complex, inter-related multiple determinants of performance. Our findings, including new factors, contribute to the global literature on factors affecting the performance of CHWs and have wide application.

## Introduction

Utilising the individual and collective potential of community health workers (CHWs) can help overcome a range of health systems challenges such as the health workforce shortage, maldistribution and programme reach, to help accelerate progress towards Universal Health Care (UHC) [1]. Operating as a unique intermediary between communities and public health systems, CHWs have been effective in delivering key maternal and child health and nutrition interventions in low and middle-income countries [2, 3]

A few systematic reviews have highlighted a broad range of factors that influence the performance of CHWs [4–6]. They have identified factors influencing performance at three levels: at the level of the individual CHW, at the level of the programme or interventions the CHW is part of, and at a broader contextual level [4, 5, 7, 8]. These reviews reiterate that there is a need to investigate the contextual factors and enablers (how, for whom, under what circumstances), and the broader health system requirements [9–11]. This connects to the theoretical literature situating CHWs as operating both within complex adaptive health systems heavily influenced by system ‘software’ (i.e. norms, values, incentives, relationships and power dynamics) as well as within heterogeneous communities that themselves resemble complex, adaptive systems [8, 12]. Understanding the factors that act as facilitators and barriers to performance requires research that gains in-depth insights into the realities of the lives of CHWs and the communities they serve.

**Fig 1:**
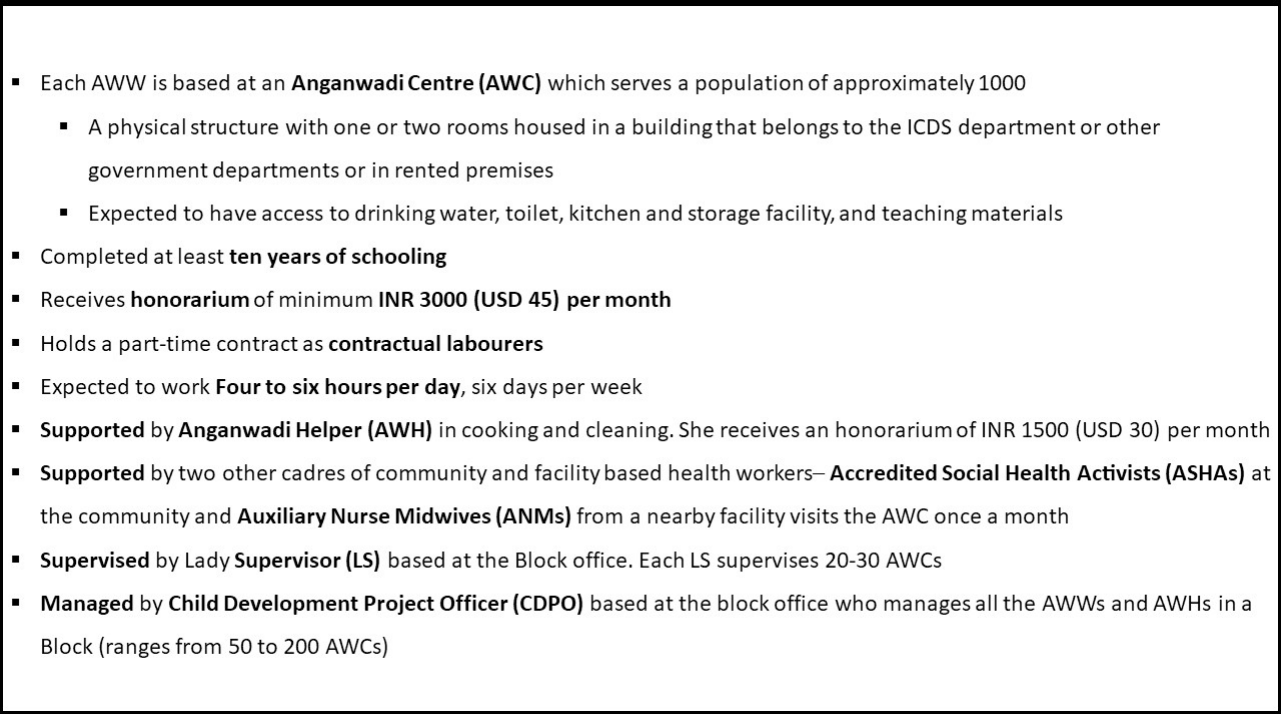
AWWs' working arrangements.

In this paper, we examine the factors affecting the performance of workers within one of the world’s largest CHW programmes [13]. India’s Integrated Child Development Services (ICDS) scheme employs 1.34 million village nutrition workers–Anganwadi Workers (AWWs) [14]. AWWs are responsible for delivering key preventive public health and nutrition services to women and children including food supplements in the form of Take Home Rations (THR), nutrition and health education, pre-school education, supporting the health system to provide health check-ups and immunisation at the monthly Village Health Sanitation and Nutrition Day (VHSND), and referral services. The services AWWs deliver can be categorised as product–oriented services (such as food supplements or vaccines) and information–oriented services (such as individual and group counselling) [15]. A summary of AWWs’ working arrangements are given in **Fig 1**.

The literature suggests that the ICDS and AWWs have significant potential to deliver health and nutrition interventions and that the utilisation of services has increased in the last decade [16, 17]. However, the utilisation of services varies across Indian states and there remains considerable room for improvement [18]. For example, in Bihar, India’s poorest state, only 37.2% of pregnant women, 35.0% of breastfeeding women, 54.0% of children under 3 years, and 43.4% of children aged between 3 and 6 receive any services from AWWs [18]. This suggests that a large proportion of the eligible population are either excluded or are opting out from the AWW service delivery net.

Considering AWWs are primarily responsible for the delivery of ICDS services, their performance is critical for the overall programme performance [19]. However, the evidence base on what shapes AWW performance is limited. The evidence on the ICDS that is available primarily focuses on describing the shortfall in human resource management (workload, incentives, role clarity, and supervision) and resources and logistics (job aids, transport, and supplies), but not on the actual worker [16, 20, 21]. Moreover, there has not been a systematic analysis of the broader range of factors identified in the global literature, and few studies that examine facilitators and barriers to performance from AWWs’ perspectives, particularly how they manage the interface with the complex community systems that they are themselves part of [9, 22, 23]. Our study aims to fill this evidence gap by exploring AWWs’ perceptions of *what* and *how* individual, programme, community, and organisational factors influence their performance. Learning about the factors influencing the performance of these workers has the potential to support efforts to strengthen service delivery in India whilst also generating potential lessons for other large scale global CHW programmes.

## Materials and methods

A qualitative study of 30 AWWs in the Indian state of Bihar was conducted to understand factors that influence their performance.

### Conceptual framework and definition of performance

**Fig 2:**
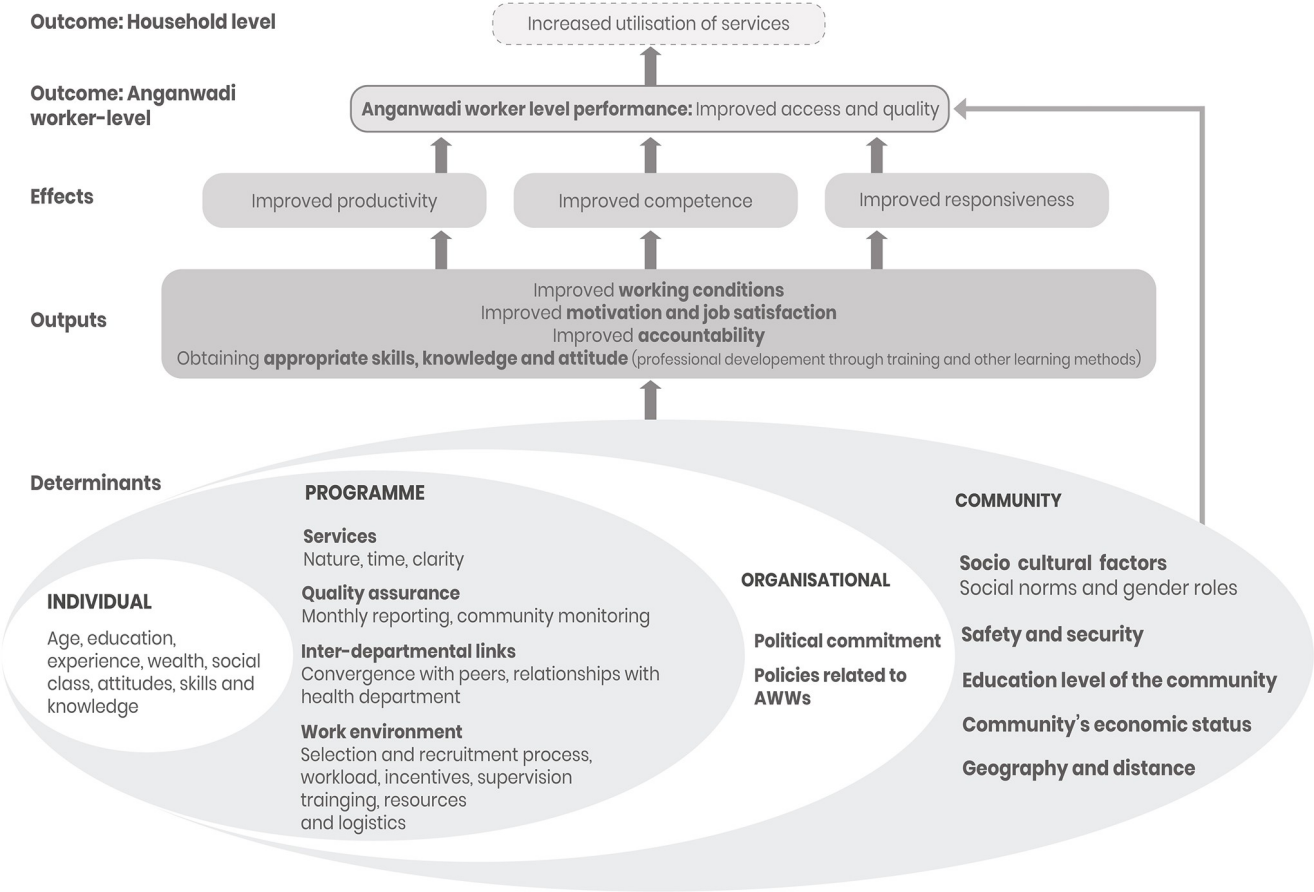
Conceptual framework on AWW performance.

We adapted previous conceptual frameworks pertaining to CHW performance to the AWW context [4, 7, 8]. We used the conceptual framework as an analytic instrument to guide the research and organise the results (see **Fig 2**).

This study defines AWW performance as ‘*AWWs making services accessible with required quality (i*.*e*. *fidelity to guidelines)’*. The determinants of AWW performance are categorised as individual, programme, community, and organisational. The factors illustrated in the framework are identified in previous systematic reviews [4, 5]. The determinants of performance are expected to be interdependent and lead to improvements in outputs such as working conditions, motivation, job satisfaction, accountability, and AWW’s skills, knowledge, and attitudes. These outputs are further expected to lead to effects such as improved productivity, competence, and responsiveness of the AWWs and lead to performance outcomes at the AWW level.

Table 1 describes the performance outcomes of each of the services provided by the AWW. For example, AWWs are expected to provide food supplements based on a fixed number of beneficiaries per beneficiary group as per the state government’s guidance and not as per their eligibility (e.g. 4 kilograms of rice and 2 kilograms lentils for 12 severely malnourished children). AWWs are expected to adhere to these guidelines on types of beneficiary groups, number of beneficiaries per group, and quantities and types of food groups.

**Table 1:** Access and quality elements of services provided by the AWW (source [24, 25]).

Core services	Performance outcomes		
	Quality Activities to be performed within each of the services and its frequency		Access Number of beneficiaries to be served per service
**Pre-school**	• Engage in pre-school learning activities • Record attendance • Annual disbursement of uniform funds	Daily	40; Children 3 to 6 years
**Supplementary Nutrition**	• Daily hot cooked meal as lunch • Includes eggs once a week • Purchase raw materials • Record keeping of purchase and distribution	Daily	40; Children 3 to 6 years
	• Monthly dry THR • Identification of beneficiaries based on malnutrition status • Distribute pre-decided quantity of rice and lentils for each beneficiary category • Record keeping of purchase and distribution	Monthly	8; Pregnant women
			8; Lactating mothers
			40; Children 6 months to 3 years: (28 severely malnourished and 12 malnourished)
**Immunisation**	• Provide space for VHSND • Help the ANM to carry out the VHSND at the AWC premises • Ensure age-appropriate immunisation and reporting • Inform beneficiaries, upkeep of immunisation due lists	Monthly	All; Pregnant women Children below two years
**Health Check-ups**	• Weighing of pregnant women and children • Counselling and record keeping	Monthly	All; Pregnant women, children below three years
	• Weighing of pre-school children • Counselling of parents and record keeping	Quarterly	40; Children 3–6 years
**Health and Nutrition Education**	• Lead group counselling on health, nutrition and sanitation themes	Weekly	All; Women, adolescent girls
	• Home visits for one-to-one counsellling on individual needs such as breastfeeding, complementary feeding, etc.	Daily	2–3 per day; All pregnant women, lactating mothers and children below two years
**Referral**	• Identification and referral of SAM children	Any time	All; SAM children

### Selection of sites and participants

We conducted the study in two administrative blocks of one of the districts in the state of Bihar, India. Demographic characteristics were collected from a random sample of 110 AWWs, from which 30 were later purposively selected using three criteria: i) Distance to the block office from the Gram Panchayat (GP) ─ a significant distance an AWW needs to travel regularly for official purposes and could influence supervisory visits; ii) Education of the AWW─a key recruitment criterion; and iii) Caste of the AWW ─ which could affect the overall trust she receives from the community and the trust she creates by either perpetuating or challenging caste divisions through her own service delivery actions [15, 26]. Our sample included AWWs from all distance, education, and caste categories.

In India, different caste groups are categorised into caste categories for administrative purposes. This categorisation reflects a traditional hierarchy. For example, that there are higher caste groups such *Brahmans* and *Kshatriyas* that are categorised in the General category; a group of the traditionally most excluded castes and tribal peoples categorised respectively as Scheduled Caste (SC) and Scheduled Tribe (ST), and those in between, the Other Backward Class (OBC). These are categories reflecting historical disadvantages and are important for various measures of redressal such as quotas for state education and employment.

### Data collection and analysis

Semi-structured interviews with AWWs were conducted in the local language (Hindi) in September 2015. The topics covered during the interviews included reasons for taking up the job, workload, supervision, training, community, incentives, and facilitators and barriers in delivering the core ICDS services. Interviews were conducted alone with participants at their homes or at the AWC by the first author or a female research assistant. The first author interviewed half of the participants, and the female research assistant conducted the rest. The first author and the female research assistant did the initial introduction together as the latter was a good entry point for an outsider to get to know the AWW and her social relationships. The first author listened to all interviews conducted every day by the female research assistant to check whether the interviews were of expected depth. All interviews were audio-recorded with the permission of the AWWs, then transcribed verbatim, and translated into English. Each interview lasted for 45–60 minutes. Two note-takers teamed up with the first author and the female interviewer to record extensive field observations for all interviews.

A hybrid method of inductive and deductive thematic analysis was used to analyse the data to combine deductive analysis guided by the conceptual framework on AWW performance and inductive analysis with the generation of new emerging themes from the data [27, 28]. As the first stage of analysis, the transcripts were uploaded and coded with the help of the qualitative data management program NVivo 10 [29]. Once the interviews were fully coded by the first author, a second-stage coding and summarising for emergent themes and patterns was conducted.

#### Ethics approval

The ethics committee at the University of Sussex, UK gave the ethics approval for the larger mixed methods doctoral research conducted by the first author. The institutional review board at the International Food Policy Research Institute (IFPRI), USA and Centre for Media Studies, India provided ethics approval for this specific qualitative study. Informed consent was obtained from study participants prior to interview.

## Results

Table 2 provides a summary of demographic characteristics of AWWs. The majority of AWWs were aged between 31−40 years, had at least 12 years of education, and belonged to the OBC category. In both blocks, AWCs either had their own buildings or operated from other governmental buildings.

**Table 2:** Anganwadi workers’ characteristics.

Characteristics	Block A	Block B
**Age**		
Up to 30 years	5	6
31–40 years	9	8
41–50 years	1	1
**Education**		
10 years of education	4	3
12 years of education	8	6
Graduate	3	5
Post-graduate	0	1
**Caste**		
General	4	4
Other Backward Caste	9	8
Scheduled Caste	2	3

**Table 3** summarises the range of individual, programme, community, and organisational factors identified and how they influence AWW performance. Some factors affects specific services and others affect overall job performance. Although the categories are the same as in the conceptual framework, some factors identified in the study had not been previously described in the literature to be influential on AWW performance.

**Table 3:** Summary of findings: Factors influencing AWW performance.

Category	Factors	Previously identified in the literature as influential in AWW performance? (Yes/No)	Which services are affected by the factor?	Does the factor affect specific outcomes? (Yes/No)	Which outcomes does the factor affect? (Access, quality or both)	How does the factor affect? (Facilitator or Barrier)
**Individual**	*Financial motive*	No	All job	No		
	*Family support*	No	Food distribution	Yes	Access	Facilitator
**Programme**	*Service preference of beneficiaries and AWWs*	No	Food distribution	Yes	Access	Facilitator
			Immunisation	Yes	Access	Facilitator
			Pre-school	Yes	Access	Facilitator
	*Work environment Workload*	Yes	All job	No		
	*Honorarium*	Yes	All job	No		
	*Supervision*	Yes	All job	Yes	Access Quality	Facilitator/ Barrier
	*Resources Human*: *helper*	Yes	Pre-school Food distribution	Yes	Access Quality	Facilitator
	*Material*: *supplies*	Yes	Food distribution Pre-school	Yes	Access Quality	Barrier
	*Material*: *job aid*	Yes	Immunisation	Yes	Access Quality	Facilitator
	*Material*: *infrastructure*	Yes	Pre-school Food distribution	Yes	Quality	Barrier
	*Financial*	Yes	Food distribution	Yes	Access Quality	Barrier
			Pre-school	Yes	Quality	Barrier
**Community**	*Caste dynamics of the AWW and community*	No	All job	Yes	Access Quality	Barrier
	*Seasonal migration*	No	Food distribution	Yes	Access	Barrier
**Organisational**	*Corruption*	No	Food distribution	Yes	Access Quality	Barrier

### Individual factors

#### Financial motive rooted in family income needs

AWWs discussed three types of initial motives (financial, moral, and social) to take up the job. Whilst some AWWs actively chose to take up the role due to ‘moral’ motives (the opportunity to positively impact their community), for all there was a strong ‘financial’ motive to support their families: the salary (Rs. 3000 (USD 45) per month) was a key source of household livelihood. Therefore, the financial motive was more important than the social and moral motives, both for taking up the role but also for continued motivation.

In some cases, AWWs fought against the familial and social pressures that prevented her from working outside the home. In these cases too, they chose to work as an AWW to contribute to the family income and secure their children’s future. In one of the interviews, a graduate AWW who works predominantly in an SC settlement echoed the opinion of many AWWs by saying:

“Reason is that see everybody, even every woman wants to do something now, and they do not want to sit idle at home as earlier women used to do. Now we are getting educated, and for the sake of our children, we have to do it. Then poverty is also a reason as we are very poor.” [AWW Interview_24]

### Family support

For many AWWs, their families decided to apply for the job on their behalf after a brief consultation with them. Their families tended to be more focused on the ‘financial’ and ‘social’ motive–the additional income and prestige within the community and access to social and bureaucratic networks that came with the job. In most cases, an AWW’s access to resources and power remained entangled with the family because she lacked an independent identity in the village setting.

A majority of AWWs stated that their family members, especially their husbands, supported their daily job responsibilities, particularly in procuring materials for food preparation, filling monitoring forms, and providing transport. AWWs whose husbands were deceased are either helped by sons or other male family members as most of them lived in joint families. Support from the family acts as a facilitator for the AWW to make services such as food distribution accessible (performance outcome) to beneficiaries. One of the AWWs who takes help from her husband for her work said:

“Both husband and wives are involved in this job. This Anganwadi is not meant for one. It cannot be done unless two people are there. When the rice comes, my husband has to go and bring it. It is very heavy, and a woman cannot lift it. So for that, I send my husband. […] So out of 3000, only 1500 is given to the worker and 1500 to her husband. So what does one get?” [AWW Interview_13]

### Programme factors

#### Service preference of beneficiaries

A majority of AWWs perceived that beneficiaries preferred product–oriented services (for example food or vaccines) to information–oriented services (that involve counselling). AWWs perceived that beneficiaries’ explicit preference for products attracted better attendance for the THR distribution and immunisation compared to the individual and group counselling sessions. The majority of AWWs felt that this occurred due to the beneficiaries’ perception of an AWW as a source of benefit and the belief that they should receive a tangible benefit for their time. AWWs often mentioned that while counselling during home visits, the beneficiaries said: “*you do not give us anything* [referring to food] *and keep telling us the same things*”.

AWWs perceived that women were unwilling to participate in group discussions as AWWs discontinued providing refreshments during the sessions due to budget cuts. Consequently, fewer beneficiaries began to attend the sessions. A few AWCs even discontinued the sessions. One AWW explained the beneficiary preference for the product–oriented services:

“We do not need to tell them much the day we distribute egg, rice or dress. The day money is distributed; there is no need to tell more than once. However, on the day of Mahila Mandal (group counselling) and immunisation, we have to call them several times. Some lie and say that they cannot come they have to go somewhere. We feel very bad, and many times we get angry as well and say that “on the day of distribution you all come running. When we call you to say something, then you all don’t come”. [AWW Interview_14]

“Yes, we do have Mahila Mandal (group counselling). It happened in 2007 and it continued a few years later. We used to receive Rs 150 for women to distribute tea and snacks. […] ANM used to give us. But after that as once Mahila Mandal got closed (funds stopped) and it is still closed. No one is interested in doing it again”. [AWW Interview_30]

The AWWs’ perception that beneficiaries prefer product-oriented services has a mixed influence on AWW performance. This encourages the AWWs to make product–oriented services such as food distribution, immunisation and pre-school accessible but hinder the provision of information–oriented services like home visits and group counselling sessions.

#### Service preference of AWWs

Even when food was not available, AWWs reported that they provided pre-school teaching. A few AWWs stated that they distribute snacks to keep children interested in the pre-school activities when food was unavailable. Although this could be due to strict supervision, detailed exploration revealed AWW’s own preference towards the pre-school service. The majority of AWWs self-identified themselves as teachers rather than as nutrition workers. AWWs stated that they liked pre-school services the most, as it gave them the status of a teacher for children. A teacher’s job remains as an available, achievable and respectable position for educated women in villages. In the interviews, the majority of AWWs added that they had always aspired to become teachers but were not able to get positions as teachers. A quote by an AWW who used to be a literacy programme facilitator is a good example revealing the initial aspiration to become a teacher: *“What else could I do*, *I filled the form here and got selected*. *I came for the teacher only*. *Later on*, *when the vacancy came for a teacher’s post*, *I was short of money”*. [AWW Interview_03]

#### Work environment

Factors such as workload, honorarium, supervision, and resources in the work environment of AWWs were found to influence their performance.

*Workload*. The majority of AWWs interviewed felt overburdened and emphasised that the high workload demotivated them and influenced job satisfaction, especially as they felt that the government did not pay them an adequate wage for their effort. AWWs compared their workload with that of other frontline workers in the village and listed different types of population surveys as additional work that they have to take on:

“[……] we do feel that we are the only ones who are burdened with all the work. We have been made a multi-purpose worker. The entire load comes to us. In a village, AWW is there, then the ward members, the village head, ASHA, ANM, doctor, BDO (Block Development Officer), CEO (Chief Executive Officer), etc. However, the entire burden is on us only”. [AWW Interview_18]

*Honorarium*. All AWWs discussed low and delayed honoraria as a major demotivating factor in the work environment. They complained that, despite being an educated workforce, their pay remained lower than the daily wage rate of unskilled workforce. Moreover, AWWs also received their monthly honorarium with three to four months of delay. The feeling of not being valued by the state government hinders an AWW’s motivation and self-esteem:

“These days, a labourer also brings Rs. 200 in the evening as a day’s wage. Are we not worth more than a labourer? Are we not worth being a labourer even after doing graduation? We were sad when we did not get increment this time. This hurt not only me but all the Sevikas (AWWs)”. [AWW Interview_03]

*Supervision*. Supervisors who assisted AWWs in their tasks (i.e. helped the worker in convincing reluctant beneficiaries to vaccinate their children, explained monitoring checklists, formats, and registers, and helped with the THR distribution) positively affected AWWs performance. Punitive supervision focused on auditing compliance and delivered in a hierarchical manner demotivated AWWs. An AWW recollects how a supervisor’s behaviour evoked fear and damaged the purpose of providing feedback or task assistance: “*What a voice she [supervisor] has*! *[*…*]*, *if there was a mistake she used to explain in such a bad manner […]*. *If there is a person*, *who has such a voice that one would fear and think that if one has to ask something then how should we ask*?*”*

The demotivation increased especially when supervisors were acting as a channel for organisational corruption (i.e. when a supervisor takes a share from the money that AWWs are meant to use to procure food for beneficiaries—discussed in a later section).

*Resources*. AWWs suggest that three types of resources influence their performance: human, material, and financial. Table 4 summarises findings on different resources and how these factors influence AWW performance.

**Table 4:** Summary of work environment resource factors.

Work environment factor: Resources	Factors	How these factors influence performance	AWW quotes
**Human**	Anganwadi Helper	Facilitates performance by helping to improve the access and quality elements of pre-school service	*Whenever meeting is there*, *our madam informs us*. *CDPO madam asks us to tell my helper (sahayika) to inform the children that today we will not go to play games*. *I tell her to cook the meal before 1 o’clock and I give helper (sahayika) all the necessary things to cook the meal*. *I instruct her that tomorrow we have a meeting in our office so I would not come and so you would cook the mid-day meal on time*, *distribute the poshahar (mid-day meal) among the children on time and leave them on right time as well*. *After that I take my register and go to the office*. *(AWW Interview_29)*
**Material** ***Supplies***	Erratic availability, inadequate quantity, and low quality of rice supply	Impedes to performance by demotivating the AWW and impacting on the access and quality elements of food distribution	*There is a problem in THR as well*. *Like government has sent us 50 kg*. *But it turns out to be 40 or 42 or 45 also*. *So we have to manage and give what we get*. *We tell them that 250gms is less*. *How can we give 2 and half kilos when it is less in the bag*? *This was we inform everyone and manage*.*[…] They say that you get 3 kilos but give 2 ½ kilos*. *So we say no*. *I say that I will weigh it in front of you*. *So I get it weighed in front of them and show them the amount of rice in the sack*. *We manage accordingly and distribute*. *[AWW Interview_15]*
***Job aid***	Job aids such as immunisation due-list register	Facilitates the quality element of immunisation service by helping the AWW to adhere guidelines	*If name is there in the due list*, *whoever gets the vaccine we write their names in the register*. *Register is maintained for children from 6 months to 2 years*. *So we take the register where their name is mentioned*. *[…] We tick against the names*. *We write who has got the polio*, *DPT1*, *DPT2 etc*.*[…] [AWW Interview_18]*
***Infrastructure***	Lack of building	Hinders AWW motivation and performance by influencing the access and quality elements of pre-school teaching	*We face problems because the centre is running at the community building*. *It would have been better if we had our own centre*. *Because there is no place*, *we are not able to give place*. *Those who are from there say that “it is our building then where should we go to sit*, *you are asking us to move*. *You build your own centre*.*”[AWW Interview_04]*
**Financial**	Inadequate and erratic funds (for food and uniform)	Facilitates AWW motivation and performance by causing rifts in the community and affecting the access and quality elements of food distribution and pre-school	*We don't get the money for pulse according to the market rate*. *That time we tell Madam that*, *dal is Rs*.*80 or Rs*. *90 and we get Rs*. *55 from the government*. *So from the beginning it's expensive*. *Dal rate is anyway expensive*.*[AWW Interview_10]*

The only additional human resource support to the AWW, the Anganwadi Helper, cleans the AWC, prepares food, and brings the children to the AWC. Her presence facilitates AWW performance by helping to improve the access and quality elements of pre-school services.

The erratic availability, inadequate quantity, and low quality of rice supply influence an AWW’s ability to deliver the service as prescribed by the guidelines. These issues strain the AWW’s relationship with the community because people frequently suspect that the AWW deliberately avoids food distribution. This demotivates an AWW and may contribute to adversely affect the access and quality elements of AWW performance outcomes.

The AWC infrastructure, especially the unavailability of AWC buildings with a kitchen area, storage (to store food items, utensils, registers, and teaching aids), and water facility (for cooking and cleaning children) affected AWWs’ workload and hindered the quality of pre-school services. AWWs who used communal spaces in the villages due to the lack of AWC buildings had to deal with anti-social behaviour and harassments by drunkards and continuous damage to the mud stove. This also demotivates AWWs and hinders the quality of pre-school services.

The immunisation due-list register acts as a job aid for the worker to adhere to guidelines and therefore enhances the quality of services. Although AWWs considered the number of monitoring and reporting registers burdensome, they felt the immunisation due-list registers helped them to identify children for age-appropriate immunisation.

AWWs receive two types of financial resources—a) a monthly fund (Rs. 11,000/ $165) to procure and distribute food except for rice (inclusive of transport allowance) and b) an annual uniform fund (Rs. 250/ $3.75) per child) to distribute among forty preschool children. The monthly fund to procure and distribute food is pre-fixed by the government, i.e. a certain amount of money is budgeted for each of the raw materials such as *dal* (lentil), vegetables and eggs. This leaves AWWs to cope with inflation, which they manage with difficulty by giving beneficiaries less than the required amount of food. Moreover, AWWs do not receive these funds on time, which causes further disruptions. In the case of uniform funds, the money is given for forty children per AWC and creates rifts with the community as the community demands uniform money for all children in the family. This clash of expectation even caused even episodes of physical fights with AWWs and community members.

### Community factors

#### Caste dynamics

In a few interviews, caste dynamics between the AWW and community, and discrimination towards the AWW, emerged as factors influencing AWW performance. AWW catchment areas tended to cover multiple caste groups which made it challenging to satisfy all groups. Higher caste communities do not always welcome services from lower caste AWWs, and higher caste group AWWs found it challenging to satisfy lower caste groups and members of her own caste group. This is aggravated when resources are limited, and AWWs could not cover all beneficiaries, leading to perceptions of unfairness and erosion of trust.

For example, in one of the cases where an AWW belonged to the OBC and worked in a predominantly SC (*Manjhi*) hamlet; she faced conflicts with *Manjhis* and other higher caste groups (*Rajputs*, *Yadavs*). Due to the strict ceiling for food and other material benefits (uniform money for children), she could not either cover all *Manjhi* families or all from other caste groups. This led to perceptions of bias, physical violence episodes and created a hostile environment prohibiting the worker from doing her job:

“[…..] Villagers are trying hard to throw me out. They say that someone else should come at my place. Rajput people do not like a poor person working like that, and that think that I should be thrown away just like that […..]. They think, and the villagers tell me, to distribute the things among the people of my caste (general caste) only. However, whatever I am getting, that is not sufficient for them. If I am getting things for eight pregnant women, then 14–15 pregnant women would come for it. Now they say, forcefully that give us the Poshahar (supplementary food) or else give us money”. [AWW Interview_11]

In another example, the AWW belonged to a lower caste sub-group (*Ravidas*) compared to the majority of the community (*Paswan*). The conflicts started right from the recruitment stage, and the worker was banned from running the centre and faced physical violence multiple times:

AWW: Yes, then people came and said… I cannot do flag hoisting [on the Republic Day celebrations], they beat my husband and me, but some people said you do the flag hoisting.

Interviewer: What was the reason for beating you again? [This was the third time she reported the incidence of violence during the interview]

AWW: This is a Paswan Tola. When the recruitment happened, a Ravidas was chosen. I belong to Ravidas. Those people said that in a Paswan tola only a Paswan could be there not a Ravidas. “Paswan is cooking, and a Chamar (Ravidas) is sitting in the chair”… so this is what they said regarding the recruitment. [AWW Interview_07]

#### Seasonal migration

Seasonal migration by low income and lower caste groups during the monsoon months emerged as a factor hindering AWW performance. Many low-income families migrate to brick kilns every year for a few months. Seasonal migration was perceived as a challenge to the AWW as it complicated the delivery of food and other benefits. The strict demarcation of the AWC catchment area and ceiling on food distribution meant that when a family migrates out, they are no longer eligible to receive the food. In that case, AWWs replaced them with other families. However, when the low-income families return from the kilns, they needed to be re-introduced to the list and this created frictions in community dynamics.

### Organisational factors

#### Corruption

A few AWWs disclosed that they had given money to intermediaries (middlemen) during the selection process to get the job and that they pay a monthly cut (Rs. 500 to 2000) to supervisors, which they understand is shared with others in the ladder of hierarchy. AWWs pay monthly cuts from the financial resources they receive to procure and distribute food. AWWs also indicated that they received less than the required quantity of rice per sack (35–40 instead of 50 kgs) because there is pilferage during the transport to AWCs. Receiving less than the required quantity meant not distributing the mandated food entitlements to beneficiaries. However, AWWs are forced to report that they have been allocated the required quantity. These forms of corruption adversely influence AWWs’ motivation and force her to not adhere to guidelines on distributing food entitlements. A few AWWs also disclosed that they use the THR rice and lentils for their home use. Although AWWs defend their act of pilferage as a ‘consumption smoothing’ activity due to low and delayed remuneration, it undermines service delivery and performance and adversely affects the AWW’s image with beneficiaries and strains relationships.

## Discussion

Our study examined what and how individual, programme, community, and organisational factors influence AWW performance from their perspectives to understand how they manage the interface with the complex community systems that they are themselves part of.

By focusing on AWWs' perspectives about factors that influence their performance, the results capture complex mechanisms by which AWWs negotiate being both a programme worker and a community member. The findings offer a deeper understanding of the interaction between AWW's personal and professional worlds and demonstrates that the blurring of this boundary is often a coping strategy of the AWW. They seek support from family, helpers and in a few instances from higher officials. The main constraints to performance are factors outside of their control. These mainly include unavailability of programme resources to meet arbitrary targeting expectations related to food distribution, and relationships shaped by caste dynamics, seasonal migration, and corruption.

The results overall show that whilst demotivation is a strong feeling, it is the initial financial motive to take up the job which also retains them as they do not have alternative income-earning opportunities. However, it is evident that the financial motives of AWWs are not fully satisfied by their honorarium. It is the combination of the initial motive of the worker (the need to retain the job for financial needs rooted in family income needs) with the community’s expectations (for product-oriented services) which ensure continued efforts even when her motivation is low.

The categories we presented as influential on AWW performance are same as in the conceptual framework (see Fig 2). However, some factors identified in the study had not been previously described in the literature (e.g. financial motive, family support, service preference, caste, seasonal migration, and corruption), some are in line with the framework (e.g. workload, honorarium, supervision, and resources) and the rest that were in the framework (Fig 2) did not arise.

Our results highlight three key empirical contributions in understanding the performance of CHWs such as AWWs.

Firstly, the AWW perception of beneficiary preference towards product-oriented services and her own preference towards pre-school education offers a unique empirical understanding on how an AWW manages her diverse service delivery basket and her latent biases towards certain services. Although a few studies have pointed out the identity clashes that AWWs feel, the results of this study contribute a rich analytical account of how her self-identity as a pre-school teacher augments her motivation and favours the delivery of pre-school and food distribution and impedes the delivery of information-oriented counselling services [30, 31]. The results also highlight the unique ways in which beneficiary preferences for product-oriented services significantly shapes an AWW’s performance. The NFHS-4, (2015) confirmed this pattern of service delivery as it reported 33% pregnant women received THR but only 18% received any nutrition or health counselling. Although other studies confirm the low coverage of services in Bihar, this study adds to the existing literature to provide deeper insights into what explains low coverage on the ground [16].

Secondly, our study findings show the intricate ways in which caste dynamics operate in the AWW context and hamper inclusive service delivery. The caste dynamics at the community level and migratory patterns of low-income households coupled with resource targeting impede AWW performance as the AWW could never satisfy all caste groups with the limited resources available. A few existing studies examined the influence of caste on the working of ASHAs and AWWs [31, 32]. The studies that examined caste in the ICDS context as a factor in achieving inclusive service delivery support the finding of this study [19, 33, 34]. These studies did not look at how caste influences AWW performance; instead, they discussed the role of caste in shaping the service delivery experience of the provider (AWW) and the beneficiary (community members).

Finally, studies that examined the performance of community and facility-based health workers did not identify corruption as a factor that could influence performance. Although they recognised that factors within the formal health system policy and practice are influential on performance, the dynamics of bureaucratic practices and politics is often considered as part of the local context and remains inadequately explored [32]. A few studies on the ICDS have highlighted practices of corruption in the ICDS, but did not report evidence for it or explore its relationship with AWW performance [30, 31, 33–35]. The results of this study emphasise that corruption hampers the performance of AWWs and contributes towards a deeper understanding of the embeddedness of corruption in community-level health and nutrition service delivery.

Our study findings on the negative influence of low honorarium, high workload, lack of supplies, infrastructure, and financial resources and positive influence of job aids such as immunisation due-lists corroborate the existing empirical evidence on CHW performance and the ICDS [4, 5, 20].

The mixed influence of supervision on AWW performance augments the existing evidence on supervision and performance that supportive supervision can improve performance, but punitive supervision can be counterproductive[4]. The role of the family support, often underexplored, also emerged in the AWW context as a facilitator in her performance. It extends the existing evidence on the influence of household duties on community-level workers’ performance [36, 37].

Surprisingly, training did not emerge from the AWW interviews as a factor they perceived as a facilitator or barrier in their performance. A study by Kosec et al. (2015) in Bihar also confirms this. They found that training was not a significant predictor of delivery of product-oriented services [15]. However, they take a cautionary approach by attributing it to the historically poor record of training in rural India.

The concept of trust, which is highlighted in the literature as important in determining CHW relationships with communities, is salient in our findings and it implicitly cuts across findings especially on caste, seasonality-induced migratory patterns, corruption and the perception of beneficiary preference towards products [38]. AWWs did not explicitly articulate the concept of trust in their interviews, perhaps because they focused on the factors that strained it.

In addition to the findings highlighted, which have some longer term implications for programme redesign, a key finding that has immediate policy implications is the role of seasonal migration. In addition to contributing towards providing a better understanding of the relationship between meta factors such as migration on worker performance, this finding also implies that the low-income families—especially those belonging to lower caste groups seem to be excluded from health and nutrition services while they migrate out, and are not easily re-accommodated when they return. Considering these households continue to experience poor standards of living and limited access to health systems, the state government needs to invest in concentrated efforts towards improving their lives. As a short-term solution, to better link them to AWC services, these families could be catered to using mobile AWC services or by making provisions for them to register in AWCs near their workplaces.

To address this in the long-term, it is crucial to address the issue of strict targeting that only the government of Bihar follows in India. The findings from this study suggest that the targeting not only hampers the AWW’s service specific performance, but it also hampers her community relationships, putting immense strain on the AWW. Although food distribution poses a multitude of challenges, the perception of beneficiary preference for products suggest that the AWWs will continue to need tangible products (food or non-food) to sustain beneficiary interest. In addition, the findings also underline the need for the state and national governments to significantly focus on improving the work conditions of AWWs by addressing their income needs, adequacy of resources, and identity of the AWW by aligning the workers’, programme’s and community’s expectations.

Followed by the updated national nutrition strategy [39], India launched a new campaign against malnutrition, called POSHAN Abhiyaan. A fulcrum of the new efforts is to strengthen the ICDS by supporting the capabilities of the AWWs through technological solutions and stronger training, and even some financial incentives. Our research was conducted prior to the launch of the mission; we anticipate that these insights could be helpful to the mission, especially in Bihar, but also recommend that research on the factors that motivate India’s largest workforce for nutrition be expanded to assess whether the rejuvenated efforts have their desired impacts on worker motivation and programme performance.

This research is limited by several factors. First, the interviews were conducted by the first author (AJ) and a female research assistant; hence there are possibilities of interviewer biases. However, we worked closely with each other, listened to the audio and de-briefed every day to mitigate the possible bias. Secondly, the small geographical sample could affect the generalisability of findings within the state and country. However, the findings presented in this paper corroborates the sub-optimal service delivery of the ICDS in Bihar [18]. The findings are contributing to the understanding of AWW performance by deeply capturing the voices and experiences of AWWs which are not well represented in the ICDS literature and contribute towards filling the knowledge and evidence gaps around how to support CHW programmes at scale.

## Conclusions

This paper finds the following factors as influential in AWW performance: individual factors including initial financial motive and family support; programme factors including beneficiaries’ and AWW’s service preference and work environment; community factors including caste dynamics and seasonal migration; and organisational factors including corruption. These include newly emergent factors that influence AWW performance not previously identified in the literature, which generates lessons for other contexts. The results capture complex mechanisms by which AWWs negotiate being both a programme worker and a community member. The findings offer a deeper understanding of the interaction between AWWs' personal and professional worlds and demonstrates that the blurring of this boundary is often a coping strategy of AWWs. They seek support from family, helpers and in a few instances from higher officials. The main constraints are factors outside of their control. These mainly include unavailability of programme resources to meet arbitrary targeting expectations related to food distribution, and relationships shaped by caste dynamics, seasonal migration, and corruption. Programme interventions to improve performance (such as incentives, working conditions and supportive human resource management) need to consider the complex, inter-related multiple determinants of performance.

## Supporting information

S1 File (PDF)
