# Supplementary material for: Factors influencing the performance of community health workers: A qualitative study of Anganwadi Workers from Bihar, India
Source: PLoS One. 2020 Nov 25;15(11):e0242460. doi: 10.1371/journal.pone.0242460 (PMC7688170; doi:10.1371/journal.pone.0242460)
Supplement: S1 File — (PDF) [file pone.0242460.s001.pdf]

Anganwadi worker in-depth interview
आंगनवाड़ी कार्यकर्ता से विस्तारपूर्वक साक्षात्कार

IDENTIFICATION (Please fill information A to G before the start of the interview)<br>पहचान (कृप्या इंटरव्यू शुरू करने से पहले A से G तक जानकारी भरे)				
A. Name of interviewer इंटरव्यूअर का नाम				
B. Name of note taker नोट टेकर का नाम				
C. Name of village गांव का नाम ग्राम				
D. Name of gram panchayat पंचायत का नाम				
E. Name of block ब्लॉक का नाम				
F. Name of district जिला का नाम				
G. Date तारीख	DD/MM/YYYY<br>दिन/महीना/साल			
H. Interview start and end time<br>इंटरव्यू के शुरू और समाप्त होने का समय	hrs<br>घंटे<br>Mnts<br>मिनट	hrs<br>घंटे<br>Mnts<br>मिनट		
I. Name of AWW आंगनवाड़ी कार्यकर्ता का नाम				
J. Name of AWC आंगनवाड़ी सेंटर का नाम				
K. AWC Code आंगनवाड़ी कोड				

Namaste! My name is ———————————— I represent the International Food Policy Research Institute (IFPRI). We are conducting a study to understand Anganwadi workers perceptions on factors influence their every day job responsibilities. We would like to know what motivates you, what are some factors help you do your job and difficulties you face in running the centre. The interview will take about 1 hour.
Whatever information you provide will be kept strictly confidential and will not be shown to other persons. To protect your privacy, we will keep your information using a code number rather than by your name. Only research staff will be allowed to look at your interview. Your name or other facts that might point to you will not appear when we talk about this study or publish its results.

This is for research purposes only and there are no right or wrong answers to the questions. There is no direct benefit for participating in the study today. However, the information we would gather would yield important insights about factors influencing in an Anganwadi worker's daily job responsibilities. Participation in this study is voluntary and if you choose to participate, you may withdraw at any time. Your participation or non-participation in the interview will not affect you in any way. However, I hope that you will take part in this study since your participation is important.

If you have questions during the interview, ask at any time. If you have any additional questions about the study you may contact: Aparna John, IFPRI, 9th Floor, Aggarwal Towers, Rajendra Place, New Delhi-110008 Phone: + 91-11-66219282. If you have any ethical issues regarding this study, you may contact: CMS IRB, Research House, Saket Community Centre, New Delhi 110 017 Phone:+91- 11 2685 1660/2685 6429.

नमस्ते! मेरा नाम __ हैं। मैं इंटरनेशनल फ़ूड पॉलिसी रिसर्च इंस्टीट्यूट (IFPRI) से हूँ। हम आंगनवाड़ी कार्यकर्ताओ से उनकी दैनिक जिम्मेदारियों को प्रभावित करने वाले कारको के बारे में उनकी धारणा समझने के लिए एक स्टडी कर रहे हैं। हम जानना चाहते हैं कि आपको कौन सी चीज़े प्रेरित करती हैं, कौन से कारक आपके कार्य पुरे करने में आपकी मदद करते हैं और केंद्र चलाने में आपके सामने कौन सी समस्याएं आती हैं। साक्षात्कार में लगभग 1 घंटा लग सकता हैं।

आपके द्वारा दी गई जानकारी को पूर्ण रूप से गोपनीय रखी जायेगी और यह किसी अन्य व्यक्ति को नहीं दिखाई जाएगी। आपकी निजी जानकारी भी आपके नाम की जगह एक पहचान कोड संख्या की सहायता से गुप्त रखी जायेगी। सिर्फ इस अध्ययन से जुड़े शोधकर्त्ता के अलावा किसी और को देखने की अनुमति नही होगी। जब इस अध्ययन के बारे में हम कही बाते/चर्चा करेंगे या इसकी रिर्पोट प्रकाशित करेंगे तब भी आपके नाम–पता इत्यादि को पूर्ण रूप से सुरक्षित रखा जायेगा।

यह सिर्फ रिसर्च के उद्देस्य के लिए की जा रही हैं और यहां प्रश्नो के कोई सही या गलत जवाब नहीं हैं। इस स्टडी में भाग लेना स्वैच्छिक हैं और यदि आप भाग लेते हैं तो भी आप किसी भी समय इसे छोड़ सकते हैं। इस साक्षात्कार में भाग लेना या भाग नहीं लेना आपको किसी भी तरह प्रभावित नहीं करेगा। हालाँकि मुझे उम्मीद हैं कि आप इसमें भाग लेंगे क्योकि आपकी भागीदारी हमारे लिए बहुत महत्वपूर्ण हैं।

यदि इंटरव्यू के दौरान आपका कोई सवाल हो तो आप कभी भी पूछ सकते हैं। आज आपके इस अध्ययन से सोधे तौर पर लाभ नही होगा। हालांकि आपके द्वारा दि गई जानकारी/सूचनाएं इकट्ठा कर हमे आंगनवाड़ी कार्यकर्ताओ से उनकी दैनिक जिम्मेदारियों को प्रभावित करने वाले कारको / तत्व के बारे में उनकी धारणा समझने /जानने में मदद मिलेगी ।

इंटरव्यू के दौरान यदि आपको किसी प्रकार का प्रश्न पूछना है तो आप किसी भी समय पूछ सकती है। अगर आपको इस अध्ययन के बारे कुछ और भी प्रश्न पुछना है तो कृप्या आप इनसे संर्पक कर सकते हैः अर्पणा जौन, ईफरी, नौवा तल्ला, अग्रवाल टावर्स, राजेन्द्र पलेस, नई दिल्ली – 110008, फोन नं0: + 91–11–66219282. और यदि इस अध्ययन से संबधित कोई नितिगत बातें/विषय हो तो कृपया आप इनसे संपर्क कर सकते हैंः सीएमएस, आइआरबी, रिसर्च हाउस, साकेत कम्युनिटी सेंटर, नई दिल्ली – 110 017, फोन नं0 – +91–11 2685 1661ध्2685 6429.

ANSWER ANY QUESTIONS AND ADDRESS RESPONDENT'S CONCERNS.
उत्तरदाता के सभी सवालो का जवाब दे और उनकी चिंता/शंका दूर करे।

Would you like to participate in this interview?
क्या आप इस साक्षात्कार में भाग लेना चाहते हैं?

Motivation

प्रेरणा

1. Question: How did you become interested in the work as an Anganwadi worker?
   प्रश्न: आपको आंगनवाड़ी कार्यकर्ता बनने की इच्छा कैसे हुई?

[Probes]
[प्रोब करे]
- What are some reasons for you to become AWW?
  आपके आंगनवाड़ी कार्यकर्ता बनने के क्या कारण हैं?
- How did you come to know about the opportunity to become AWW, how did you apply, get selected?
  आपको आंगनवाड़ी कार्यकर्ता बनने के अवसर के बारे में कैसे पता चला, आपने इसके लिए आवेदन कैसे किया और आपका चयन कैसे हुआ?

Workload
कार्यभार

2. **Question:** As an AWW what do you do on a typical day?
   प्रश्न: आंगनवाड़ी कार्यकर्ता के रूप में आम तौर पर आप के दिन कैसे होते हैं?

[Probes]
[प्रोब करे]

- When do you generally wake up in the morning? / How do you spend your time in the morning hours?
  सामान्यतौर पर आप सुबह कब उठते हैं?/ आप सुबह क्या काम करने में अपना समय बिताते हैं?
- When do you start your day as AWW?
  आंगनवाड़ी कार्यकर्ता के रूप में आप अपना दिन कैसे शुरू करते हैं?

2.1. **Question:** How often do you need to go for meetings/trainings/union meetings etc?
प्रश्न:आपको मीटिंग्स/ ट्रेनिंग/ यूनियन मीटिंग आदि में कब-कब जाना पड़ता हैं?

2.2. **Question:** Other than being an AWW what do you do?

प्रश्न: आंगनवाड़ी के कम के अलाव आपको ओर क्य कम होत हैं?

2.3. **Question:** How do you juggle home and work responsibilities? Was there any periods you felt difficult to balance home and work responsibilities? Please could you describe that time?

आप घर और काम संबंधी जिम्मेदारियों को कैसे मैनेज करते हैं? क्या कोई ऐसा समय भी आता हैं जब आपको घर और काम संबंधी जिम्मेदारियों को पूरा करना मुश्किल लगता हैं? कृप्या क्या आप उस समय के बारे में बता सकते हैं?

Programme responsibilities: facilitators and barriers

कार्यक्रम की जिम्मेदारियाँ: मार्गदर्षक एवं बाधाएं

3. Question: Please tell us about services you deliver as an AWW?
   प्रश्न: कृप्या आगनवाड़ी कार्यकर्ता के रूप में आपके द्वारा दी जाने वाली सेवाओ के बारे में बताये?

   (*Please listen to her carefully and make note of the services she mentioned and ask about each of the services she mentioned using the probes for each of the service.*)
   (कृप्या उन्हें ध्यानपूर्वक सुने और उनके द्वारा बताई गई सेवाओ को नोट करे और उनके द्वारा बताई गई प्रत्येक सेवा के लिए प्रोब करे।)

4. Question: Please tell us how the Supplementary Nutrition programme is carried out?
   प्रश्न: क्या आप बतायेगे कि पोशहर में आप क्या क्या करते हैं?

[Probes]
[प्रोब करे]

— *If she mentioned that she serves* Hot Cooked Meal (HCM), *please ask:* You mentioned earlier that you serve —— children HCM daily. Could you please tell me how is this carried out in a daily basis? (*discuss* ***raw material procurement, finances, transportation, storage, cooking, serving, and cleaning***)

  यदि उन्होंने बताया हो कि वह बच्चों को **"गर्म पका खाना" (HCM)** देती हैं तो कृप्या पूछे: आपने मुझे पहले बताया था कि आप बच्चो को रोजाना "गर्म पका खाना" (HCM) देती हैं। क्या आप मुझे यह बता सकती हैं कि प्रतिदिन यह कार्य किस तरह से किया जाता है। (कच्चा माल कैसे खरीदती हैं. **रूपये-पैसे का प्रबंध कैसे करती हैं. परिवहन की व्यवस्था. भंडारण की व्यवस्था, खाना पकाना, खाना परोसने की व्यवस्था एवं सफाई की व्यवस्था इत्यादि कैसे करती हैं आदि के बारे में चर्चा करे**)

  - Do you experience any problems with HCM distribution? If yes, what types of problems? (Probe separately raw material procurement, finances, transportation, storage, cooking, food quality, serving, and cleaning)

    क्या आपको "गर्म पका खाना" (HCM) बांटने में किसी समस्याओं का सामना करना पड़ता हैं? यदि हाँ. किस प्रकार की समस्याओं का सामना करना पड़ता हैं?(कच्चा माल प्राप्त करने. रूपये-पैसे का प्रबंध करने, परिवहन की व्यवस्था. भंडारण की व्यवस्था. खाना पकाना, खाना परोसने की व्यवस्था एवं सफाई की व्यवस्था इत्यादि के लिए अलग-अलग प्रोब करे)

— *If she mentioned that she serves* ***Take Home Ration (THR)****, please ask:* You mentioned earlier that you serve —— children and mothers THR monthly. Could you please tell me how is this carried out on a monthly basis?
यदि वह बताती है कि वह ”घर ले जाने के लिए आहार“ (THR) देती है तो कृप्या पूछें कि: आपने पहले बताया था कि आप बच्चों एवं माताओं को हर महीने ”घर ले जाने के लिए आहार“ (THR) देती है।क्या आप मुझे यह बता सकती हैं कि हर महीने यह कार्य किस तरह से किया जाता है?

- Do you experience **any problems with THR distribution**? If yes, what types of problems? (Probe separately **THR procurement, finances, transportation, storage, identification of beneficiaries, THR quantity per beneficiary, THR quality**)
  क्या ”घर ले जाने के लिए आहार“(THR) बांटने में आपको किसी समस्याओं का सामना करना पड़ता हैं?यदि हाँ, तो आपको कौन सी समस्याओं का सामना करना पड़ता हैं? (THR प्राप्त करने, **रूपये-पैसे का प्रबंध कैसे करती हैं, परिवहन की व्यवस्था, भंडारण की व्यवस्था, लाभार्थी की पहचान करने में, प्रत्येक लाभार्थी को मिलने वाली THR की मात्रा,THR की क्वालिटी आदि के बारे में चर्चा करे**)

— *If she says she doesn't serve THR or HCM, please ask:* You mentioned that you don't serve children THR or HCM lately. What are the problems you experience in the food distribution (Probe separately for THR and HCM)

यदि वह बताती है कि अब वह THR या HCM नहीं देती तो कृप्या पूछें कि: आपने बताया कि अब आप बच्चों को THR या HCM नहीं देती। आपको खाने का वितरण करने में कौन-कौन सी समस्याओ का अनुभव करना पड़ा था?(THR और HCM के लिए अलग-अलग प्रोब करे)

5. **Question:** Please tell us how the **growth monitoring sessions** are carried out at your AWC
प्रश्न: आप्के केन्द्र में वज़न निगरानी किस तरह् किया जाता हैं?

[Probes]
[प्रोब करे]

- Did you ever experience problems with the weighing scale? If yes, what kinds of problems? What do you usually do if this happens? Do you have an extra scale?
  क्या कभी आपको वज़न करने वाली मशीन के साथ कोई समस्या हुई हैं? यदि हाँ, किस प्रकार की समस्या? यदि ऐसा होता हैं तो आमतौर पर आप क्या करते हैं? क्या आपके पास एक्स्ट्रा स्केल/मशीन होती हैं?

- What do you do after you weigh children? (Probe about growth charts and counselling to mothers) What do you do after you weigh mothers?
  बच्चे का वजन लेने के बाद आप क्या करती हैं? (ग्रोथ चार्ट के बारे में तथा माँ को सलाह् देने के बारे में प्रोब करे) गर्भवती महिलाओ का वजन लेने के बाद आप क्या करती हैं?

- What do you feel about conducting growth monitoring?
  वजन निगरानी करना आपको कैसे लगता हैं?

- What are some factors help you conduct growth monitoring?
  बच्चो के विकास की देखरेख करने संबंधी सेशन का आयोजन करने में आपकी मदद करने वाले कौन-कौन से कारक हैं?

- What are some problems you face in weighing children and women? (Probe about difficulties in actual measurement process, recording of data, understanding the cases of growth falter, if a woman doesn't bring her MCP card how do you record her weight and how do understand whether she had any weight gain, and counselling mothers)
  बच्चों और महिलाओं का वजन लेने में आपको किन समस्याओं का सामना करना पड़ता है?(वास्तविक माप प्रक्रिया, डाटा को रेकार्ड / लिखने, विकास/ग्रोथ के उपर-नीचे होने वाले केस समझने, अगर महिला एम0सी0पी0 कार्ड लेकर नहीं आती है तो आप वज़न कैसे रेकार्ड करते हैं तथा आप कैसे समझते हैं कि महिला का वजन बढ़ा है या नहीं, और फिर सलाह् कैसे देते हैं के बारे में अलग-अलग प्रोब करे)

6. **Question:** Please tell us about the **Immunization services** at your AWC
   प्रश्न: आपके आंगनवाड़ी केंद्र पर **टीकाकरण सेवा** कैसे होता हैं?

[Probes]
[प्रोब करे]

- What is your role in providing immunization services?
  टीकाकरण दिवस में आपकी क्या भूमिका होती हैं?

- How do you feel about the coordination between ASHA and ANM in conducting the immunization?
  टीकाकरण दिवस का आयोजन करने में आशा और एएनएम के बीच में कोर्डिनेशन/सामंजस्य के बारे में आप क्या महसूस करते हैं?

- What are some problems you face in conducting the immunization day (probe separately about coordination with ASHA and ANM, availability of vaccines, availability of MCP cards)
  टीकाकरण दिवस का आयोजन करने में आपको कौन-कौन सी समस्याओ का सामना करना पड़ता हैं (आशा और एएनएम के बीच में कोर्डिनेशन/सामंजस्य; टीको, एमसीपी काड्र्स की उपलब्धता, के बारे में अलग से पूछे)

7. **Question:** How does the Village Health and Nutrition Day (VHND) work at your center?
   प्रश्न: आपके आंगनवाड़ी केंद्र पर ग्रामीण स्वास्थ्य और पोषण दिवस (वीएचएनडी) कैसे काम करता हैं?

- What all services are provided at the VHND? How often the VHND is carried out?
  ग्रामीण स्वास्थ्य और पोषण दिवस (वीएचएनडी) में कौन कौन सी सेवाएं दी जाती हैं? ग्रामीण स्वास्थ्य और पोषण दिवस (वीएचएनडी) का आयोजन कब-कब किया जाता हैं?

- What is your role in the VHND?
  ग्रामीण स्वास्थ्य और पोषण दिवस (वीएचएनडी) में आपकी क्या भूमिका होती हैं?

- What are some factors help you do your part at the VHND? ग्रामीण स्वास्थ्य और पोषण दिवस (वीएचएनडी) में अपनी भूमिका निभाने में आपकी मदद करने वाले कौन-कौन से कारक हैं?

- What are some problems you face in conducting the VHND (probe separately about coordination with ASHA and ANM, availability of vaccines, IFA tablets, ORS sachets, Vitamin A

syrup, Paediatric anaemia syrup, and deworming tablets, availability of MCP cards, availability of weighing machines)

ग्रामीण स्वास्थ्य और पोषण दिवस (वीएचएनडी) का आयोजन करने में आपको कौन-कौन सी समस्याओ का सामना करना पड़ता हैं (आशा और एएनएम के बीच में कोर्डिनेशन/सामंजस्य: टीको. आईएफए टैबलेटस. ओआरएस के सैशे. विटामिन ए का सिरप. बच्चो में खून की कमी के लिए सिरप और डीवॉर्मिंग टैबलेट्स की उपलब्धता, एमसीपी काड्र्स की उपलब्धता, वज़न करने की मशीन की उपलब्धता के बारे में अलग से पूछे)

8. **Question:** Please tell us how you do **home visits**?
   प्रश्न: आप ग्रुह् भेंट के दौरान क्य क्य होते हैं?

[Probes]
[प्रोब करे]

- How many home visits you conduct every month? How frequently you do this?
  हर महीने आप कितने ग्रुह् भेंट करते हैं? आप ऐसा कब-कब करते हैं?

- What do you discuss during home visits?
  आप ग्रुह् भेंट के दौरान क्या चर्चा करते हैं?
  - What do you discuss with pregnant mothers, newly delivered mothers, and mothers of young children?
    आप गर्भवती महिलाओ. हाल ही में जन्म देने वाली नई माताओ और छोटे बच्चो की माताओ से क्या चर्चा/बातचीत करती हैं?

- What are some problems you face in conducting home visits (probe separately about planning, understanding about counselling, beneficiaries responsiveness etc.)
  ग्रह भेंट करने में आपको कौन-कौन सी समस्याओ का सामना करना पड़ता हैं (योजना बनाना, काउंसलिंग के बारे में समझना, लाभार्थियों की प्रतिक्रिया आदि के बारे में अलग से पूछे)

9. **Question:** Please tell us how you carry out **pre-school** activities
प्रश्न: कृप्या हमे बताये कि आप **स्कूल** पूर्व शिक्षा में क्या क्या करते हैं?

[Probes]
[प्रोब करे]

— What are some problems you face in conducting pre-school activities (probe separately about lack of toys and other resources, familiarity with curriculum, number of children, private schools etc.)
प्री-स्कूल संबंधी गतिविधियों का आयोजन करने में आपको कौन-कौन सी समस्याओ का सामना करना पड़ता हैं (खिलौनों और अन्य संसाधनो, पाठ्यक्रम के बारे में जानकारी, बच्चो की संख्या, प्राईवेट स्कूल आदि के बारे में अलग से पूछे)

10. **Question:** Do you conduct any group **nutrition and health education** activities at your center? If so, what kind of group sessions you conduct?
प्रश्न: आप के सेन्टर में क्या **पोषण और स्वास्थ्य शिक्षा संबंधी** चर्चा होती हैं? आप किस प्रकार ये चर्चा करते हैं?

[Probes]
[प्रोब करे]

- On an average how many sessions do you conduct in a month? What do you discuss in those sessions?
हर महीने आप कितने पोषण और स्वास्थ्य शिक्षा संबंधी सेशन का आयोजन करते हैं? आप इन सेशन में क्या चर्चा करते हैं?
- What are some problems you face in conducting these sessions (probe separately participant's interest, availability of job aids, time constraints etc.)
इन सेशंस का आयोजन करने में आपको कौन-कौन सी समस्याओ का सामना करना पड़ता हैं (भागीदारों का रूचि लेना, कार्य में सहायता की उपलब्धता, समय आदि के बारे में अलग से पूछे)

11. **Question:** We'd like to know how you feel about your responsibilities as an AWW at present.
प्रश्न: आंगनवाड़ी क कम आपको कैसा लगता हैं?

[Probes]
[प्रोब करे]

- What do you like about working as AWW?
  आंगनवाड़ी कार्यकर्ता के रूप में काम करने के बारे में आपको क्या पसंद हैं?
- What are some activities you do as AWW that you would like to do more often?
  आंगनवाड़ी कार्यकर्ता के रूप में आप ऐसी कौन-कौन सी गतिविधिया करती हैं, जिन्हे आप बार-बार करना चाहती हैं?
- What do you dislike the about working as an AWW?
  आंगनवाड़ी कार्यकर्ता के रूप में काम करने के बारे में आपको क्या नापसंद हैं?
- What are some activities you do as AWW that you wish you did not have to do?
  आंगनवाड़ी कार्यकर्ता के रूप में आप ऐसी कौन-कौन सी गतिविधिया करती हैं, जिन्हे आप नहीं करना चाहती हैं?
- Why do you want to work as an AWW?
  आंगनवाड़ी कार्यकर्ता के रूप में आप कम क्यों करना चाहती हैं?

Community

समुदाय

12. **Question:** How do you feel about working in this community?
    प्रश्न:आपको इस समुदाय में काम करना कैसा लगता हैं?

[Probes]

[प्रोब करे]

- How does being an AWW affect your social status in the community?
  आंगनवाड़ी कार्यकर्ता बनने से समुदाय में आपकी सामजिक स्थिति किस प्रकार प्रभावित होती हैं?
- What do you think others in the community think about you?
  आपको क्या लगता है कि समुदाय आप के बारे में क्या सोच्ते है?

TBGI/ BCSP (only for programme AWWs)

TBGI/ BCSP (सिर्फ कार्यक्रम में शामिल आंगनवाड़ी कार्यकर्ताओ के लिए)

13. **Question:** What are your thoughts about the ___ programme?
    प्रश्न: आपको ___प्रोग्राम कैसा लगता हैं?

[Probes]

[प्रोब करे]

- What kind of support you are receiving to promote the program?
  आपको प्रोग्राम को प्रमोट करने के लिए किस प्रकार का समर्थन मिलता हैं?
- How does the programme influence your daily responsibilities?
  यह प्रोग्राम आपकी दैनिक जिम्मेदारियों को किस प्रकार प्रभावित करता हैं?
- How did you do (service) before the programme? How has it changed now?
  प्रोग्राम से पहले आप यह (सेवा) कैसे करते/देते थे? अब यह कैसे बदल गया हैं?

Training

प्रशिक्षण

14. **Question:** How do you feel about the training that you have received this year?

प्रश्न: आपको इस साल मिला प्रशिक्षण कैसा लगा?

[Probes]

[प्रोब करे]

- How do you feel about the training that you received first time?
  आपको पहली बार मिला प्रशिक्षण कैसा लगा?

- Are there are aspects of your job responsibilities that you feel you need more/better training for?
  क्या आपकी कार्य संबंधी जिम्मेदारियों के कुछ ऐसे पहलू हैं,जिनमे आपको प्रशिक्षण मिलने/बेहतर प्रशिक्षण मिलने की जरूरत लगती हैं?

Supervision
सुपरविज़न/निरीक्षण

15. **Question:** How do you feel about the supervision structure in the program?
    प्रश्न: आपको प्रोग्राम का सुपरविज़न स्ट्रक्चर कैसा लगता हैं?

[Probes]
[प्रोब करे]

- Who all supervise your work? (Probe ICDS supervisors and non-ICDS monitors)
  आपके काम का निरीक्षण/सपवीज़न कौन-कौन करता हैं? (प्रोब करे आईसीडीएस सुपरवाइज़र्स और नॉन आईसीडीएस सुपरवाइज़र्स)
- Do you feel that you receive adequate supervisory support when you have problems, when you need to plan, when you need technical advice, etc.?
  क्या आपको लगता हैं कि जब आपको कोई समस्या होती हैं, कोई योजना बनाने की जरूरत होती हैं, किसी तकनीकी सुझाव की जरूरत होती हैं आदि में आपको पर्याप्त सुपरवाईज़री सपोर्ट मिलता हैं?
- Do you get along with your supervisor? What's something you would like to change in your relationship with the supervisor?
  क्या आपका सुपरवाइज़र आपके साथ होता हैं? आप अपने और अपने सुपरवाइज़र के बीच संबंधो में क्या बदलाव करना चाहते हैं?

Demographic information
जनसांख्यिकीय जानकारी

AWW's Age
आंगनवाड़ी कार्यकर्ता का उम्र
No. of adults in the house:
आपके परिवार में कुल कितने व्यस्क सदस्य हैं?
No. of children in the house:
आपके परिवार में कुल कितने बच्चे हैं?
Main source of income for the household:
आपके परिवार का आय का मुख्य श्रोत/ साधन क्या है?
Highest level of education of the AWW:
आंगनवाड़ी कार्यकर्ता ने कहाँ तक पढ़ाई पूरी की हैं?
Religion of AWW:
आंगनवाड़ी कार्यकर्ता का धर्म
Caste of AWW:
आंगनवाड़ी कार्यकर्ता की जाति
Caste category of AWW: SC/ST/OBC/General
आंगनवाड़ी कार्यकर्ता की जातिगत श्रेणी: अनुसूचित जाति, अनुसूचित जनजाति, अन्य पिछड़ी जाति या सामान्य
Main caste categoory of the AWC catchment area:
आंगनवाड़ी केंद्र के अंतर्गत आने वाली मुख्य जातिगत श्रेणी

Observations

अवलोकन
